# An evaluation of the results of convalescent plasma therapy applied to pregnant women diagnosed as COVID-19-positive in a pandemic center: A prospective cohort study

**DOI:** 10.55730/1300-0144.5346

**Published:** 2022-02-27

**Authors:** Seyit Ahmet EROL, Atakan TANAÇAN, Hakan APAYDIN, Şule GÖNCÜ AYHAN, Deniz OLUKLU, Derya UYAN HENDEM, Serpil ÜNLÜ, Abdulsamet ERDEN, Özlem MORALOĞLU TEKİN, Ahmet OMMA, İhsan ATEŞ, Orhan KÜÇÜKŞAHİN, Dilek ŞAHİN

**Affiliations:** 1 Department of Obstetrics and Gynecology, Division of Perinatology, Turkish Ministry of Health Ankara City Hospital, Ankara, Turkey; 2 Department of Internal Medicine, Division of Rheumatology, Turkish Ministry of Health Ankara City Hospital, Ankara, Turkey; 3 Department of Infectious Diseases and Clinical Microbiology, Turkish Ministry of Health Ankara City Hospital, Ankara, Turkey; 4 Department of Obstetrics and Gynecology, University of Health Sciences, Turkish Ministry of Health, Ankara City Hospital, Ankara, Turkey; 5 Department of Internal Medicine, Turkish Ministry of Health Ankara City Hospital, Ankara, Turkey; 6 Department of Internal Medicine, Division of Rheumatology, Yıldırım Beyazıt University, Turkish Ministry of Health Ankara City Hospital, Ankara, Turkey; 7 Department of Obstetrics and Gynecology, Division of Perinatology, University of Health Sciences, Turkish Ministry of Health Ankara City Hospital, Ankara, Turkey

**Keywords:** Clinical characteristics, convalescent plasma, COVID-19, laboratory parameters, pregnancy outcomes

## Abstract

**Background/aim:**

Convalescent plasma (CP) might be an additional treatment modality in COVID-19. The aim of this study was to compare CP-related clinical characteristics and perinatal outcomes in pregnant women with mild or moderate-severe COVID-19.

**Materials and methods:**

This prospective cohort study included 36 pregnant women (12 mild and 24 moderate-severe), who underwent CP therapy. The CP obtained from recently recovered donors was transfused to patients together with maximum supportive care and antiviral agents. The groups were then compared in respect of clinical characteristics, laboratory parameters, obstetric complications, and neonatal outcomes.

**Results:**

Significant differences were determined between the groups in respect of systemic corticosteroids in COVID-19 treatment (41.7%, 87.5%, p = 0.004), oxygen (O2) support (0%, 91.7%, p < 0.001), chest imaging (41.7%, 58.3%, p = 0.02), intensive care unit admission (0%, 20.8%, p = 0.03) and length of hospitalization (5.5 versus 9.5 days, p < 0.001). The O2 saturation levels before and after administration of CP were significantly lower in the moderate-severe COVID-19 group (p < 0.05). The O2 therapy time before and after administration of CP and total O2 therapy time were significantly lower in the mild COVID-19 group (p < 0.05). Platelet, plateletcrit and lymphocyte counts were significantly higher in both the mild and moderate-severe COVID-19 groups after treatment compared to the pretreatment values (p < 0.05).

**Conclusion:**

Although data on the results of CP treatment in pregnant women are somewhat limited, it has been suggested that early CP treatment may be associated with improvements in laboratory and ventilatory parameters in pregnant women with mild and moderate-severe COVID-19. Nevertheless, there is a need for further, randomized controlled studies on this subject with the inclusion of greater numbers of patients.

## 1. Introduction

At the end of 2019, an outbreak of novel Severe Acute Respiratory Syndrome Coronavirus 2 (SARS CoV-2) spread throughout the world resulting in the global Coronavirus Disease 2019 (COVID-19) pandemic [1]. To date, certain antivirals such as the malaria drug hydroxychloroquine, remdesivir, lopinavir/ritonavir, and favipiravir have been used in the treatment of COVID-19. For critically ill patients, in addition to supportive therapy, such as oxygen supply and extracorporeal membrane oxygenation (ECMO), various therapies such as cytokine inhibitors, stem cell therapy, and immune convalescent plasma are still being investigated [2]. Convalescent plasma (CP) is not available in routine practice and has not yet been licensed by the United States Food and Drug Administration (FDA). However, it has been made available for specific pathogens during periods of epidemic or pandemic. After a minimum of 2 weeks of full recovery from COVID-19, individuals who meet the necessary criteria can donate CP^1^. This CP obtained from recovered COVID-19 patients provides immunity based on passive antibodies. Although neutralizing antibodies are thought to be the main active compounds, other immune mediators in plasma may also contribute. It is thought that CP which contains high neutralizing antibody titers may have clinical benefits when administered early in the course of the disease [3]. Moreover, it has been reported that CP therapy applied in addition to antiviral therapy in severe adult COVID-19 patients can improve viremia and other clinical indices [4]. During the COVID-19 pandemic, over 100,000 units of CP therapy have been administered as part of an expanded access program, with an emergency use permit or during clinical trials^2^.

There are studies in the literature that have reported the successful administration of CP on a few pregnant women, sometimes in conjunction with medical therapy (e.g., steroids, remdesivir, lopinavir/ritonavir, and azithromycin) [5–7]. However, the data regarding the efficacy and safety of CP therapy in pregnant women are insufficient^3^. The research into the effects of CP on COVID-19 patients is ongoing and is open to pregnant women who meet the inclusion criteria^4^. Furthermore, there are few studies in the literature evaluating the maternal-fetal, neonatal results, and related laboratory parameters of pregnant women treated with CP.

The aim of this study was to compare CP-related clinical characteristics, obstetric complications, maternal-fetal and neonatal outcomes in pregnant women with mild or moderate-severe COVID-19, and to determine the association of these factors with laboratory parameters.

## 2. Materials and methods

### 2. 1. Study design and case selection

This prospective cohort study included 36 pregnant women, who were applied with CP therapy in the Department of Obstetrics and Gynecology of Ankara City Hospital between 28 August 2020 and 12 October 2020. Pregnant women between the ages of 18 and 40 years were included in the study. Approval for the study was granted by both the Turkish Ministry of Health and the Institutional Ethics Committee (E2-20-24). Informed consent was obtained from all the patients. Ankara City Hospital is a Ministry of Health tertiary reference center, which has played a major role in the management of pandemic patients since March 2020, when the first COVID-19 case was recorded in Turkey [8]. For pregnant women who are diagnosed with COVID-19, there are special protocols with follow-up carried out by a multidisciplinary team, comprising obstetricians, perinatologists, chest diseases specialists, infectious diseases specialists, rheumatologists, anesthesiologists, and neonatologists. Although Ankara City Hospital is a pandemic center, the follow-up of all pregnant women continues, resulting in approximately 1100 deliveries per month [9].

The diagnosis of COVID-19 was made from the real-time polymerase chain reaction (RT-PCR) assay applied to nasopharyngeal and oropharyngeal specimens [10]. National guidelines were followed for the classification of the severity of disease and management of COVID-19 patients^5^. The COVID-19 convalescent plasma (CP) obtained from recently recovered donors was transfused to patients at a dose of 200 mL high neutralizing titers (>1/80) together with maximum supportive care and antiviral agents. CP therapy was not administered to patients with immunoglobulin-A (IgA) deficiency^3^. The CP administered to each patient came from a single batch.

### 2. 2. Study parameters

The pregnant women undergoing CP treatment in this study were classified into two groups as mild and moderate-severe. The groups were then compared in respect of demographic features, clinical characteristics, obstetric complications, and neonatal outcomes. Maternal age, body-mass index (BMI) (kg/m^2^), gravidity, parity, previous miscarriage, comorbid conditions, gestational age at diagnosis, pregnancy status, route of delivery, indications for cesarean delivery, obstetric complications, tocolytic therapy, antenatal corticosteroid therapy for fetal maturation, COVID-19 treatment, oxygen (O2) support, chest imaging, intensive care unit (ICU) admission, length of hospitalization, birth weight, gestational age at birth, 1 and 5-min Apgar scores, and neonatal intensive care unit (NICU) admission rates were compared between the groups.

CP-related clinical characteristics such as the day of plasma administration, allergic reaction and side-effects after administration of plasma, O2 saturation and O2 therapy time before and after administration of plasma, total O2 therapy time, body temperature, heart rate, systolic blood pressure, and diastolic blood pressure before and after administration of plasma were compared between the pregnant women with mild and moderate-severe COVID-19.

The CP-related laboratory parameters of initial white blood cell (WBC), neutrophil, and lymphocyte counts, neutrophil-lymphocyte ratio (NLR), C-reactive protein (CRP), interleukin 6 (IL-6), procalcitonin, ferritin, platelet (PLT), plateletcrit (PCT), alanine transferase (ALT), aspartate transferase (AST), D-dimer, fibrinogen, and immunoglobulin A (IgA) levels were compared before and after administration of plasma between pregnant women with mild and moderate-severe COVID-19.

The laboratory parameters and clinical characteristics such as fibrinogen, ferritin, procalcitonin, CRP, PCT, WBC, PLT, neutrophil, lymphocyte, NLR, AST, ALT, IL-6, D-dimer, O2 saturation, body temperature, heart rate, systolic and diastolic blood pressure before and after the administration of CP were compared between the groups of pregnant women with mild and moderate-severe COVID-19.

### 2. 3. Statistical analysis

Data obtained in the study were analyzed statistically using SPSS vn. 26.0 software (Statistical Package for the Social Sciences for Windows, IBM Corpn, Armonk, NY, USA). Descriptive statistics were presented as median and interquartile range values as the variables did not show normal distribution. Median values were compared between groups using the Mann-Whitney U-test. Categorical variables were reported as number (n) and percentage (%). The chi-square test was applied to the comparisons between groups of categorical variables. The Wilcoxon signed-rank test was used in the comparison of dependent groups. The level of statistical significance in all the analyses was accepted as a two-tailed value of p < 0.05.

## 3. Results

CP treatment was administered to 12 (33.3%) pregnant women with mild COVID-19 and to 24 (66.6%) pregnant women with moderate-severe COVID-19. CP therapy is more preferred in the moderate-severe group in COVID-19. The comparisons between the mild and moderate-severe groups in respect of demographic features, clinical characteristics, obstetric complications and neonatal outcomes are shown in Table 1. Significant differences were determined between the groups in respect of systemic corticosteroids in COVID-19 treatment (41.7%, 87.5%, p = 0.004), O2 support (0%, 91.7%, p < 0.001), chest imaging (41.7%, 58.3%, p = 0.02), ICU admission (0%, 20.8%, p = 0.03), and length of hospitalization (5.5 versus 9.5, p < 0.001, respectively). Other variables were similar in the two groups.

Comparisons between pregnant women with mild and moderate-severe COVID-19 in respect of CP-related clinical characteristics are shown in Table 2. The two groups were comparable in terms of the plasma administration day, allergic reaction and side-effects, body temperature, heart rate, and systolic and diastolic blood pressure values before and after administration of plasma were similar in both groups. The O2 saturation levels before and after administration of plasma were significantly lower in the moderate-severe COVID-19 group (p < 0.05). The O2 therapy time before and after administration of plasma and total O2 therapy time were significantly lower in the mild COVID-19 group (p < 0.05).

The comparisons of CP-related laboratory parameters between the mild and moderate-severe COVID-19 groups are shown in Table 3. In respect of initial WBC, neutrophil, lymphocyte counts, NLR, CRP, IL-6, procalcitonin, ferritin, PLT, PCT, ALT, AST, D-dimer, fibrinogen, and IgA levels, the two groups were comparable, and no difference was determined between the groups after CP administration in respect of lymphocyte, NLR, CRP, IL-6, procalcitonin, ferritin, PLT, PCT, ALT, AST, D-dimer and fibrinogen levels. The WBC and neutrophil levels were found to be significantly higher in the moderate-severe COVID-19 group (p = 0.03).

The comparisons of laboratory parameters and clinical characteristics before and after CP administration in pregnant women with mild and moderate-severe COVID-19 are shown in Table 4. The sampling times for the parameters were defined as the day of hospitalization (before) and the first day after CP treatment (after). No differences were determined between the groups in respect of ferritin, neutrophil counts, NLR, IL-6, and diastolic blood pressure levels. In the moderate-severe COVID-19 group, fibrinogen, CRP, and body temperature values were significantly lower than in the mild COVID-19 group (p < 0.05). WBC, AST, ALT, and O2 saturation levels were determined to be statistically significantly higher in pregnant women with moderate-severe COVID-19 (p < 0.05). Procalcitonin, D-dimer, heart rate, and systolic blood pressure levels were statistically significantly lower in the mild COVID-19 group compared to the pregnant women with moderate-severe COVID-19 (p < 0.05). PCT, PLT, and lymphocyte counts were significantly higher in both the mild and moderate-severe COVID-19 groups after treatment compared to the pretreatment values (p < 0.05).

## 4. Discussion

The main findings of the present study indicated that convalescent plasma might be an additional treatment modality in selected pregnant women. Potentially favourable effects were seen on the inflammation parameters and the necessity for oxygen therapy in both mild and severe cases. As knowledge is still limited on the efficacy and safety of convalescent plasma for pregnant women with COVID-19, these findings may be of guidance in future studies. Although the relatively low number of cases and the absence of a control group due to the unique characteristics of the study population limit the interpretation of the results, this study revealed favorable outcomes of the use of CP in pregnant women with COVID-19.

The World Health Organization (WHO) and the US Food and Drug Administration (FDA) reported that convalescent plasma (CP) was used during the H1N1 influenza virus epidemic in 2009–2010, and in the outbreaks of SARS-CoV-1 in 2003, and Middle East Respiratory Syndrome (MERS-CoV) in 2012^6^. It has also been reported that the use of CP containing antibodies against COVID-19 may be effective against this latest infection [11]. The IgG antibodies in particular, which are specific to SARS-CoV-2 and are passively transferred by the transfused plasma, are thought to have a role in neutralising viral particles and activating the complement system, thereby allowing elimination of the virus [12]. In a study of 10 patients with severe COVID-19, Duan et al. administered a single dose of 200 mL CP from recently healed donors with neutralizing antibody titres >1/640 in addition to standard therapy. The results demonstrated that within 3 days, the clinical symptoms significantly improved with increased oxyhemoglobin saturation, and various parameters showed an improving trend, including increased lymphocytes and decreased CRP. CP treatment has been reported to be well tolerated and may have the potential to improve clinical outcomes by neutralizing viremia in patients with severe COVID-19 [13]. The results of the current study showed that CRP and fibrinogen levels decreased, and O2 saturation and lymphocyte levels increased after CP treatment in the moderate-severe COVID-19 group.

For individuals who have not been previously exposed to or vaccinated with the viral pathogen, the development of the response to antibodies can take up to two to three weeks [14]. Therefore, the provision of antibodies has the positive potential of shortening the duration of the disease and preventing serious life-threatening complications [15]. However, it has been stated that it may be more appropriate to administer CP to patients with a severe disease course in the early stages of clinical admission, before the need for intensive care develops [12, 16–18]. When there is critical infection due to organ damage or massive inflammation, the benefit of CP on these complications remains uncertain [19]. During the recovery period of COVID-19, the IgM anti-Spike protein (S) and anti-Nucleocapsid protein (N) have been reported to start to appear within about a week, and continue to increase for two weeks, while IgG has been observed to increase after a few days (typically during the third week). This change has been seen to occur more rapidly in nonintensive care unit patients (ICU) compared to ICU patients [20].

Increased survival has been reported in randomized studies using high titer CP in the early stages of the disease. In a previous, randomized, double-blind, placebo-controlled study of 160 patients aged >65 years, the administration of CP within 72 h of the onset of symptoms (mild disease) was seen to result in a lower probability of progression to severe disease (respiratory rate ≥ 30/min, oxygen saturation < 93%), and a lower probability of mortality [16]. In the current study, CP was transfused to patients in both the mild and moderate-severe groups in the early period. In a randomized study conducted on 81 hospitalized patients, the requirement for high-flow oxygen, mechanical ventilation, or extracorporeal membrane oxygenation (ECMO), and the mortality rates on days 15–29 were reported to be lower for patients treated with CP (0% vs. 14%, 0% vs. 9%, respectively) [21]. In an observational study including more than 35,000 patients treated with CP in the USA, lower mortality rates (7-day mortality: 9% vs. 12%) were reported in patients administered with CP within the first 3 days of presentation [22]. Similarly, in another observational study of 351 patients treated with CP, mortality rates were lower in patients who were not intubated and received CP within 72 h of hospitalization (60-day mortality rate: 4% vs. 12.3%) [23].

In studies, which did not include sufficient antibody titer (<1/80), or in which CP treatment was administered in the late stage of the disease, no positive benefits could be seen on survival and disease progression. This was attributed to the onset of an endogenous self-antibody response within 8–10 days of infection and disease progression, in addition to the inflammatory response rather than the virus itself [24]. CP treatment appears to be applicable without delay and without disrupting other available therapies, including antiviral agents. Moreover, as there is expected to be lower efficacy of CP therapy in mutant SARS-CoV-2 variants in the receptor binding sites, there is a need for further studies [25].

Although the optimal dose of CP is still unknown, institutional or clinical trial protocols should be followed. In the current study, early and single-dose CP therapy was administered as described in literature^3^. It is also important to know that plasma transfusions may be associated with transfusion reactions such as allergic reactions, acute lung injury due to transfusion, and circulatory overload [26,27]. Approximately 20,000 CP replacements have been reported to have been concluded with low rates of negative side-effects such as transfusion reactions, thromboembolic complications, and cardiac events (<1%, 1%, and 3%, respectively) [28]. In the current study, no serious negative side-effects were observed related to the CP transfusion.

Previous studies have reported the successful administration of CP in a limited number of pregnant women, sometimes in combination with medical therapy (e.g., steroids, remdesivir, lopinavir/ritonavir, azithromycin). In a case report of a 24-week pregnant patient with COVID-19 pneumonia followed up in ICU, Grisolia et al. administered 2 doses of 300 mL CP on the 7th and 12th days after the onset of symptoms, together with medical treatment and reported rapid normalization in body temperature and SPO2, decreased CRP and improved clinical status [29]. In another case of a 35-week pregnant patient, Zhang et al. administered 300 mL of CP on day 19 in addition to medical treatment due to severe acute respiratory distress syndrome (ARDS), multiple organ dysfunction syndrome (MODS), and septic shock after COVID-19. It was concluded that CP could be a potential treatment option for critically ill COVID-19 patients [30]. Anderson et al. administered CP to a 22-week pregnant COVID-19 patient with comorbid type 2 diabetes mellitus (DM), asthma, and class 3 obesity, in addition to the medical treatment as ARDS developed 24 h after hospital admission and the patient required ICU. It was emphasized in that report that the remdesivir treatment may shorten the extubation period, and reduce mortality in patients with COVID-19 in need of mechanical ventilation [7]. In the current study, patients had similar comorbidities, with a higher incidence in the moderate-severe group. It has been reported that attentive corticosteroid therapy combined with the transfusion of CP may lead to positive results in pregnant women with ARDS in terms of suppressing viremia and cytokine storm [6]. A 27-week intubated pregnant patient, who was treated with 2 doses of CP on the 4th and 5th days of hospitalization, was discharged on the 14th day. This highlighted that the administration of CP could be associated with maternal survival, and it could be a safe alternative for pregnant women with a seronegative condition, rapid deterioration in respiratory functions, and fetal distress [5]. In accordance with the literature, increased nonreassuring fetal status, cesarean section, oxygen support and ICU admission rates were observed in the moderate-severe group in the current study.

There are few studies in literature that have evaluated the effects of CP on clinical characteristics and laboratory parameters. In a study of 25 patients with severe and/or life-threatening COVID-19, Salazar et al. stated that with CP administered at mean 6 days, there was a decrease in CRP and an increase in WBC on days 0, 7, and 14 days after the CP administration, and concluded that CP was a safe treatment option [31]. Khamis et al. reported that laboratory and ventilatory parameters improved after therapeutic plasma exchange (TPE) in 11 patients with severe COVID-19 [32]. In the current study, a decrease in procalcitonin and D-dimer levels after CP were determined in the mild group, a decrease in fibrinogen and CRP levels in the moderate-severe group, and an increase in WBC and O2 saturation, and in PCT, PLT and lymphocyte counts in both groups, consistent with previous findings in literature. The changes in WBC, AST, ALT, and O2 saturation after CP administration were determined to be statistically higher in the pregnant women in the moderate-severe group, suggesting that inflammatory processes may have increased in the moderate-severe group. However, procalcitonin, neutrophil, and IL-6 values were not statistically different in the moderate-severe group. In a study by Xia et al., it was reported that in the group with good response to CP treatment, the lymphocyte percentage was high, and neutrophil percentage and CRP were low, whereas in the nonresponsive group, lactate dehydrogenase (LDH), B-type natriuretic peptide, urea nitrogen, procalcitonin and glucose levels were found to be higher. Abnormal metabolic functions and a strong inflammatory response were associated with a low response to CP treatment [33].

Strong aspects of this study can be considered to be the prospective design with the inclusion of pregnant women compared in mild and moderate-severe groups according to several parameters. However, the study also had limitations, primarily that it was an observational study, there was no control group receiving a standard treatment regimen, and a limited number of pregnant women with COVID-19 were included. Secondly, there were no data on viral load and/or neutralizing Ab titres (in pregnant mothers and newborns).

## 5. Conclusion

Although data on the results of CP treatment in pregnant women are somewhat limited, the results of this study suggest that early CP treatment may be associated with improvements in laboratory and ventilatory parameters in pregnant women with mild and moderate-severe COVID-19. Nevertheless, there is a need for further, randomized controlled studies on this subject with the inclusion of greater numbers of patients.

## Figures and Tables

**Table 1:** Comparison of demographic features, clinical characteristics, obstetric complications, and neonatal outcomes between pregnant women with mild and moderate-severe COVID-19.

Variables	Mild COVID-19 group (n = 12)	Moderate-severe COVID-19 group (n = 24)	p value
Maternal age (years) (median, IQR)^a^	27.5 (10)	29.5 (10)	0.40
BMI (kg/m^2^) (median, IQR)^a^	24.51 (3)	25.71 (4)	0.12
Gravidity (median, IQR)^a^	2 (3)	2 (1)	0.72
Parity (median, IQR)^a^	1 (1)	1 (2)	0.79
Living child (median, IQR)^a^	1 (1)	1 (2)	0.45
Previous miscarriage (median, IQR)^a^	0 (0)	0 (0)	0.93
Comorbidity (n, %)^b^	5 (41.6%)	5 (20.8%)	0.77
Comorbidity type (n, %)^b^		0.12	
*Hypothyroidism (n, %)*	*2 (16.6%)*	*0 (0%)*	
*Thrombocytopenia (n, %)*	*1 (8.3%)*	*0 (0%)*	
*Asthma (n, %)*	*0 (0%)*	*1 (4.1%)*	
*Hypertension (n, %)*	*0 (0%)*	*2 (8.3%)*	
*Maternal cardiac disease (n, %)*	*0 (0%)*	*1 (4.1%)*	
*Diabetes mellitus type 2 (n, %)*	*0 (0%)*	*1 (4.1%)*	
*Maternal varicose veins (n, %)*	*0 (0%)*	*1 (4.1%)*	
*Hemolytic anemia*	*1 (8.3%)*	*0 (0%)*	
*Celiac disease*	*1 (8.3%)*	*0 (0%)*	
*Hydatid cyst of the liver*	*1 (8.3%)*	*0 (0%)*	
Gestational age (weeks) (median, IQR)^a^	27.5 (8.75)	26.5 (17.5)	0.32
Trimesters at diagnosis			0.38
*First trimester*	*0 (0%)*	*2 (8.3%)*	
*Second trimester*	*7 (58.3%)*	*11 (45.8%)*	
*Third trimester*	*5 (41.7%)*	*11 (45.8%)*	
Pregnancy conceived by assisted reproductive technology (n, %)^b^	0 (0%)	1 (2.7%)	
Twin pregnancy (n, %)^b^	0 (0%)	3 (8.3%)	
Pregnancy status (n,%)^b^		0.34	
*On-going pregnancy (n, %)*	*6 (50%)*	*12 (50%)*	
*Delivered (n, %)*	*6 (50%)*	*12 (50%)*	
*Route of delivery (vaginal) (n, %)* b	*3 (25%)*	*2 (8.3%)*	
*Route of delivery (cesarean section) (n, %)* b	*3 (25%)*	*10 (41.7%)*	
Indications for cesarean delivery (n, %)^b^		0.12	
*Maternal COVID-19 pneumonia (n, %)* b	*0 (0%)*	*2 (20%)*	
*Previous cesarean delivery*	*4 (100%)*	*3 (30%)*	
*Nonreassuring fetal status*	*0 (0%)*	*2 (20%)*	
*Multiple pregnancy*	*0 (0%)*	*2 (20%)*	
*Maternal sinus vein thrombosis*	*0 (0%)*	*1 (10%)*	
Obstetric complication (n, %)^b^	1 (8.3%)	6 (25%)	0.08
Obstetric complication type (n, %)^b^		0.39	
*GHT (n, %)*	*0 (0%)*	*1 (4.1%)*	
*HG (n, %)*	*0 (0%)*	*1 (4.1%)*	
*Elevated liver enzymes (n, %)*	*0 (0%)*	*3 (12.5%)*	
*Hypokalemia (n, %)*	*0 (0%)*	*3 (12.5%)*	
Tocolytic therapy (n, %)^b^	0 (0%)	1 (4.1%)	0.36
Antenatal corticosteroid therapy for fetal maturation (n, %)^b^	1 (8.3%)	2 (8.3%)	0.71
COVID-19 treatment (n, %)^b^	12 (100%)	24 (100%)	>0.05
COVID-19 treatment type (n, %)^b^			
*Hydroxychloroquine (n, %)* b	*3 (25%)*	*7 (29.1%)*	*0.79*
*Lopinavir/Ritonavir (n, %)* b	*4 (33.3%)*	*13 (54.1%)*	*0.23*
*Azithromycin (n, %)* b	*0 (0%)*	*1 (4.1%)*	*0.36*
*Favipiravir (n, %)* b	*0 (0%)*	*3 (12.5%)*	*0.10*
*Vitamin C (n, %)* b	*0 (0%)*	*1 (4.1%)*	*0.36*
*Low molecular weight heparin (n, %)* b	*11 (91.6%)*	*23 (95.8%)*	*0.60*
*Systemic corticosteroids (n, %)* b	*5 (41.7%)*	*21 (87.5%)*	***0.004***
*Anakinra (n, %)* b	*1 (8.3%)*	*8 (33.3%)*	*0.08*
*Tocilizumab (n, %)* b	*0 (0%)*	*1 (4.1%)*	*0.36*
*Remdesivir (n, %)* b	*0 (0%)*	*1 (4.1%)*	*0.37*
*Colchicine (n, %)* b	*1 (8.3%)*	*1 (4.1%)*	*0.59*
O2 support (n, %)^b^	0 (0%)	22 (91.7%)	**<0.001**
O2 support type (n, %)^b^			
*Nasal oxygen (n, %)* b	*0 (0%)*	*17 (70.8%)*	
*Reservoir mask (n, %)* b	*0 (0%)*	*2 (8.3%)*	
*High-flow (n, %)* b	*0 (0%)*	*3 (12.5%)*	
*Intubation (n, %)* b	*0 (0%)*	*1 (4.1%)*	
Chest imaging (n, %)^b^	5 (41.7%)	14 (58.3%)	**0.02**
*Chest graphy (n, %)* b	*4 (36.4%)*	*12 (50%)*	*0.45*
*CT scan (n, %)* b	*1 (8.3%)*	*4 (16.7)*	*0.47*
ICU admission (n, %)^b^	0 (0%)	5 (20.8%)	**0.03**
Length of hospitalization (days) (median, IQR)^a^	5.5 (4)	9.5 (5.5)	**<0.001**
Birth weight (gr) (median, IQR)^a^	3070 (1120)	2690 (1170)	0.13
Gestational age at birth (weeks) (median, IQR)^a^	37 (4)	37 (6)	0.40
1 min Apgar score (median, IQR)^a^	8 (1)	7 (2)	0.09
5 min Apgar score (median, IQR)^a^	9 (1)	8 (1)	0.20
NICU admission (n,%)^b^	0 (0%)	1 (8.3%)	0.33

BMI: Body-mass index, COVID-19: Coronavirus disease 2019, CT: Computed tomography, GHT: Gestational hypertension, HG: Hyperemesis gravidarum, ICU: Intensive care unit, IQR: Interquartile range, NICU: Neonatal intensive care unit, O2: Oxygen

a Statistical analysis was performed using the Mann-Whitney U test

b Statistical analysis was performed using the chi-square test

**Table 2:** Comparison of convalescent plasma-related clinical characteristics between pregnant women with mild and moderate-severe COVID-19.

Variables	Mild COVID-19 group (n = 12)	Moderate-severe COVID-19 group (n = 24)	p value
Administration day of plasma (median, IQR)^a^	2.5 (2)	4 (2.75)	0.17
Allergic reaction after administration of plasma (n, %)^b^	1 (8.3%)	0 (0%)	0.33
Side-effects after administration of plasma (n, %)^b^	3 (25%)	7 (29%)	0.40
*Fever (n, %)*	2 (16.7%)	2 (8.3%)	
*Lymphopenia (n, %)*	1 (8.3%)	2 (8.3%)	
*Fever and lymphopenia (n, %)*	0 (0%)	3 (12.5%)	
O2 saturation before administration of plasma (%) (median, IQR)^a^	98 (2)	92 (6)	**<0.001**
O2 saturation after administration of plasma (%) (median, IQR)^a^	97 (1)	94.5 (3)	**0.005**
O2 therapy time before administration of plasma (days) (median, IQR)^a^	0 (0)	2 (4)	**0.01**
O2 therapy time after administration of plasma (days) (median, IQR)^a^	0 (1.5)	4 (5.5)	**<0.001**
Total O2 therapy time (days) (median, IQR)^a^	0 (2.2)	7 (4.7)	**<0.001**
Body temperature before administration of plasma (°C) (median, IQR)^a^	37.5 (1.7)	37.4 (1.4)	0.67
Body temperature after administration of plasma (°C) (median, IQR)^a^	36.7 (0.7)	36.6 (1.4)	0.54
Heart rate before administration of plasma (per minute) (median, IQR)^a^	110 (0.2)	100 (0.2)	0.67
Heart rate after administration of plasma (per minute) (median, IQR)^a^	89 (9.5)	88 (27.2)	0.50
Systolic blood pressure before administration of plasma (mmHg) (median, IQR)^a^	117 (18.7)	110 (14)	0.31
Systolic blood pressure after administration of plasma (mmHg) (median, IQR)^a^	102.5 (11.5)	109.5 (15.5)	0.11
Diastolic blood pressure before administration of plasma (mmHg) (median, IQR)^a^	62.5 (15.7)	70 (11.2)	0.83
Diastolic blood pressure after administration of plasma (mmHg) (median, IQR)^a^	65 (10)	63 (13.2)	0.68

COVID-19: Coronavirus disease 2019, IQR: Interquartile range, O2: Oxygen

a Statistical analysis was performed using the Mann-Whitney U test

b Statistical analysis was performed using the chi-square test

**Table 3:** Comparison of convalescent plasma-related laboratory parameters between pregnant women with mild and moderate-severe COVID-19.

Variables	Mild COVID-19 group (n = 12)	Moderate-severe COVID-19 group (n = 24)	p value
WBC (10^3^/mL) (median, IQR)^a^	6430 (3315)	6630 (3773)	0.86
WBC after administration of plasma (10^3^/mL) (median, IQR)^a^	6135 (4632)	8500 (3522)	**0.03**
Neutrophil (10^3^/mL) (median, IQR)^a^	5025 (2797)	4820 (3345)	0.86
Neutrophil after administration of plasma (10^3^/mL) (median, IQR)^a^	4615 (3882)	6640 (4057)	**0.03**
Lymphocyte (10^3^/mL) (median, IQR)^a^	775 (222)	800 (442)	0.63
Lymphocyte after administration of plasma (10^3^/mL) (median, IQR)^a^	955 (285)	1150 (547)	0.68
NLR (%) (median, IQR)^a^	6.98 (4)	5.6 (4.9)	0.56
NLR after administration of plasma (%) (median, IQR)^a^	4.29 (3.8)	5.91 (6.71)	0.31
CRP (mg/dL) (median, IQR)^a^	31.1 (36.6)	45.2 (71.1)	0.50
CRP after administration of plasma (mg/dL) (median, IQR)^a^	11.2 (44.4)	21.6 (47.1)	0.58
IL-6 (pg/mL) (median, IQR)^a^	11.5 (10.3)	15.3 (13.3)	0.16
IL-6 after administration of plasma (pg/mL) (median, IQR)^a^	6.58 (8.4)	5.58 (20.4)	0.94
Procalcitonin (ng/mL) (median, IQR)^a^	0.03 (0.06)	0.03 (0.07)	0.35
Procalcitonin after administration of plasma (ng/mL) (median, IQR)^a^	0.03 (0.07)	0.03 (0.08)	0.86
Ferritin (ng/mL) (median, IQR)^a^	48 (79)	35.5 (143)	0.80
Ferritin after administration of plasma (ng/mL) (median, IQR)^a^	45.5 (107.7)	58.5 (119)	0.38
PLT (10^3^/mL) (median, IQR)^a^	172 (60.2)	189 (38.7)	0.51
PLT after administration of plasma (10^3^/mL) (median, IQR)^a^	226 (180.7)	266 (153.5)	0.26
PCT before administration of plasma (%) (median, IQR)^a^	0.15 (0.07)	0.18 (0.05)	0.62
PCT after administration of plasma (%) (median, IQR)^a^	0.21 (0.14)	0.23 (0.13)	0.31
ALT (U/L) (median, IQR)^a^	21 (10)	16 (19)	0.62
ALT after administration of plasma (U/L) (median, IQR)^a^	23.5 (32.2)	29 (87.7)	0.21
AST (U/L) (median, IQR)^a^	29 (31)	28 (26)	0.44
AST after administration of plasma (U/L) (median, IQR)^a^	38 (33.7)	35.5 (69.2)	0.67
D-dimer (mcg/mL) (median, IQR)^a^	2.1 (1.9)	2.4 (3)	0.89
D-dimer after administration of plasma (mcg/mL) (median, IQR)^a^	1.7 (0.9)	2.15 (3.48)	0.27
Fibrinogen (g/L) (median, IQR)^a^	4.4 (1.2)	4.4 (1.2)	0.51
Fibrinogen after administration of plasma (g/L) (median, IQR)^a^	4.59 (1.06)	3.98 (1.72)	0.16
IgA before administration of plasma (mg/dL) (median, IQR)^a^	1.84 (1.42)	1.48 (0.98)	0.38

ALT: Alanine transferase, AST: Aspartate transferase, COVID-19: Coronavirus disease 2019, CRP: C-reactive protein, IL-6: Interleukin 6, IgA: Immunoglobulin A, IQR: Interquartile range, PCT: Plateletcrit, PLT: Platelet count, NLR: Neutrophil to lymphocyte ratio, WBC: White blood cell

a Statistical analysis was performed using the Mann-Whitney U test

**Table 4:** Comparison of laboratory parameters and clinical characteristics before and after the administration of convalescent plasma between pregnant women with mild and moderate-severe COVID-19.

Variables	Mild COVID-19 group	Moderate-severe COVID-19 group
Fibrinogen after/before administration of plasma (z) (p value)^a^	−0.41 (0.67)	−**2.13 (0.03)**
Ferritin after/before administration of plasma (z) (p value)^a^	−1.6 (0.11)	−0.92 (0.35)
Procalcitonin after/before administration of plasma (z) (p value)^a^	−**2.05 (0.04)**	−0.79 (0.43)
CRP after/before administration of plasma (z) (p value)^a^	−1.80 (0.07)	−**2.14 (0.03)**
PCT after/before administration of plasma (z) (p value)^a^	−**2.76 (0.006)**	−**3.91 (<0.001)**
WBC after/before administration of plasma (z) (p value)^a^	−0.23 (0.81)	−**2.38 (0.01)**
PLT after/before administration of plasma (z) (p value)^a^	−**2.51 (0.01)**	−**3.92 (<0.001)**
Neutrophil after/before administration of plasma (z) (p value)^a^	−0.23 (0.81)	−1.9 (0.05)
Lymphocyte after/before administration of plasma (z) (p value)^a^	−**2.61 (0.009)**	−**2.61 (0.009)**
NLR after/before administration of plasma (z) (p value)^a^	−1.41 (0.15)	−0.54 (0.58)
AST after/before administration of plasma (z) (p value)^a^	−0.47 (0.63)	−**2.93 (0.003)**
ALT after/before administration of plasma (z) (p value)^a^	−1.41 (0.15)	−**3.45 (0.001)**
IL-6 after/before administration of plasma (z) (p value)^a^	−1.12 (0.26)	−0.85 (0.39)
D-dimer after/before administration of plasma (z) (p value)^a^	−**2.58 (0.01)**	−1.07 (0.28)
O2 saturation after/before administration of plasma (z) (p value)^a^	−1.89 (0.05)	−**2.96 (0.003)**
Body temperature after/before administration of plasma (z) (p value)^a^	−0.84 (0.39)	−**2.47 (0.01)**
Heart rate after/before administration of plasma (z) (p value)^a^	−**2.50 (0.01)**	−1.75 (0.08)
Systolic blood pressure after/before administration of plasma (z) (p value)^a^	−**2.55 (0.01)**	−0.16 (0.87)
Diastolic blood pressure after/before administration of plasma (z) (p value)^a^	−0.14 (0.88)	−1.61 (0.10)

ALT: Alanine transferase, AST: Aspartate transferase, COVID-19: Coronavirus disease 2019, CRP: C-reactive protein, IL-6: Interleukin 6, O2: Oxygen, PCT: Plateletcrit, PLT: Platelet count, NLR: Neutrophil to lymphocyte ratio, WBC: White blood cell

a Statistical analysis was performed using the Wilcoxon signed-rank test

## Footnotes

1 The United States Food and Drug Administration (FDA) (2021). Website: https://www.fda.gov/vaccines-blood-biologics/safety-availability-biologics/updated-information-blood-establishments-regarding-covid-19-pandemic-and-blood-donation [accessed on 19 January 2021]

2 Expanded Access Program for Convalescent Plasma (2021). Website: https://www.uscovidplasma.org [accessed on 28 February 2021]

3 Turkish Ministry of Health, COVID-19 Immune (Convalescent) Plasma Supply and Clinical Use Guide (2020). Website: https://shgm.saglik.gov.tr/Eklenti/39179/0/covid-19-immun-konvalesan-plazma-tedarik-ve-klinik-kullanim-rehberipdf.pdf [accessed on 30 October 2020]

4 COVID-19 Convalescent Plasma for the Treatment of Hospitalized Patients with Pneumonia Caused by SARS-CoV-2 (2021). Website: https://clinicaltrials.gov/ct2/show/NCT04397757 [accessed on 22 January 2021]

5 Turkish Ministry of Health, General Directorate of Public Health, COVID-19 (SARS-CoV-2 infection) Guideline, Scientific Committee Report (2020). Website: https://covid19.saglik.gov.tr/TR-66301/covid-19-rehberi.html [accessed on 3 September 2020]

6 The World Health Organization (WHO), Guidance on Maintaining a Safe and Adequate Blood Supply During the COVID-19↱ Pandemic and on the Collection of COVID-19 Convalescent Plasma: Interim Guidance (↱2020) ↱. Website: https://apps.who.int/iris/handle/10665/333182 [accessed on 10 July 2020]

## References

[1] Chen N Zhou M Dong X Qu J Gong F Epidemiological and clinical characteristics of 99 cases of 2019 novel coronavirus pneumonia in Wuhan, China: a descriptive study Lancet 2020 395 10223 507 513 10.1016/S0140-6736(20)30211-7 32007143 PMC7135076

[2] Lu H Drug treatment options for the 2019-new coronavirus (2019-nCoV) Bioscience Trends 2020 14 1 69 71 10.5582/bst.2020.01020 31996494

[3] Clark E Guilpain P Filip IL Pansu N Bihan CL Convalescent plasma for persisting COVID-19 following therapeutic lymphocyte depletion: a report of rapid recovery British Journal of Haematology 2020 190 3 e154 e156 10.1111/bjh.16981 32593180 PMC7361823

[4] Ciftciler R Ciftciler AE Treatment modalities in COVID 19 Aksaray University Journal of Medical Sciences 2021 2 1 20 21

[5] Magallanes-Garza GI Valdez-Alatorre C Dávila-González D Martínez-Reséndez MF Sánchez-Salazar SS Rapid improvement of a critically ill obstetric patient with SARS-CoV-2 infection after administration of convalescent plasma International Journal of Gynaecology and Obstetrics 2021 152 3 439 441 10.1002/ijgo.13467 33152797 PMC9087753

[6] Soleimani Z Soleimani A ADRS due to COVID-19 in midterm pregnancy: successful management with plasma transfusion and corticosteroids [published online ahead of print, 2020 Jul 26] The Journal of Maternal-Fetal and Neonatal Medicine 2020 1 4 10.1080/14767058.2020.1797669 32715804

[7] Anderson J Schauer J Bryant S Graves CR The use of convalescent plasma therapy and remdesivir in the successful management of a critically ill obstetric patient with novel coronavirus 2019 infection: A case report Case Reports in Women’s Health 2020 27 e00221 10.1016/j.crwh.2020.e00221 PMC7229947 32426243

[8] Sahin D Tanacan A Erol SA Anuk AT Eyi EGY A pandemic center’s experience of managing pregnant women with COVID-19 infection in Turkey: A prospective cohort study International Journal of Gynaecology and Obstetrics 2020 151 1 74 82 10.1002/ijgo.13318 32682342 PMC9087688

[9] Sahin D Tanacan A Erol SA Anuk AT Yetiskin FDY Updated experience of a tertiary pandemic center on 533 pregnant women with COVID-19 infection: A prospective cohort study from Turkey International Journal of Gynaecology and Obstetrics 2021 152 3 328 334 10.1002/ijgo.13460 33131057 PMC9087535

[10] Tanacan A Erol SA Turgay B Anuk AT Secen EI The rate of SARS-CoV-2 positivity in asymptomatic pregnant women admitted to hospital for delivery: Experience of a pandemic center in Turkey European Journal of Obstetrics Gynecology and Reproductive Biology 2020 253 31 34 10.1016/j.ejogrb.2020.07.051 32763728 PMC7390745

[11] Selvi V Convalescent Plasma: A Challenging Tool to Treat COVID-19 Patients-A Lesson from the Past and New Perspectives BioMed Research International 2020 2020 2606058 10.1155/2020/2606058 33029499 PMC7512050

[12] Rojas M Rodríguez Y Monsalve DM Acosta-Ampudia Y Camacho B Convalescent plasma in Covid-19: Possible mechanisms of action Autoimmunity Reviews 2020 19 7 102554 10.1016/j.autrev.2020.102554 32380316 PMC7198427

[13] Duan K Liu B Li C Zhang H Yu T Effectiveness of convalescent plasma therapy in severe COVID-19 patients Proceedings of the National Academy of Sciences of the United States of America 2020 117 17 9490 9496 10.1073/pnas.2004168117 32253318 PMC7196837

[14] Liu STH Lin HM Baine I Wajnberg A Gumprecht JP Convalescent plasma treatment of severe COVID-19: a propensity score-matched control study Nature Medicine 2020 26 11 1708 1713 10.1038/s41591-020-1088-9 32934372

[15] Bloch EM Shoham S Casadevall A Sachais BS Shaz B Deployment of convalescent plasma for the prevention and treatment of COVID-19 The Journal of Clinical Investigation 2020 130 6 2757 2765 10.1172/JCI138745 32254064 PMC7259988

[16] Libster R Pérez Marc G Wappner D Coviello S Bianchi A Early High-Titer Plasma Therapy to Prevent Severe Covid-19 in Older Adults The New England Journal of Medicine 2021 384 7 610 618 10.1056/NEJMoa2033700 33406353 PMC7793608

[17] Tobian AAR Shaz BH Earlier the better: convalescent plasma Blood 2020 136 6 652 654 10.1182/blood.2020007638 32761223 PMC7414595

[18] Moniuszko-Malinowska A Czupryna P Zarębska-Michaluk D Tomasiewicz K Pancewicz S Convalescent Plasma Transfusion for the Treatment of COVID-19-Experience from Poland: A Multicenter Study Journal of Clinical Medicine 2020 10 1 28 10.3390/jcm10010028 33374333 PMC7795721

[19] Roberts DJ Miflin G Estcourt L Convalescent plasma for COVID-19: Back to the future Transfusion Medicine 2020 30 3 174 176 10.1111/tme.12700 32447783 PMC7283818

[20] Sun B Feng Y Mo X Zheng P Wang Q Kinetics of SARS-CoV-2 specific IgM and IgG responses in COVID-19 patients Emerging Microbes and Infections 2020 9 1 940 948 10.1080/22221751.2020.1762515 32357808 PMC7273175

[21] Avendaño-Solá C Ramos-Martínez A Muñez-Rubio E Ruiz-Antorán B Malo de Molina R A multicenter randomized open-label clinical trial for convalescent plasma in patients hospitalized with COVID-19 pneumonia The Journal of Clinical Investigation 2021 131 20 e152740 10.1172/JCI152740B 34473652 PMC8516461

[22] Joyner MJ Senefeld JW Klassen SA Mills JR Johnson PW Effect of Convalescent Plasma on Mortality among Hospitalized Patients with COVID-19: Initial Three-Month Experience medRxiv 2020.2020.08.12.20169359 10.1101/2020.08.12.20169359

[23] Salazar E Christensen PA Graviss EA Nguyen DT Castillo B Significantly Decreased Mortality in a Large Cohort of Coronavirus Disease 2019 (COVID-19) Patients Transfused Early with Convalescent Plasma Containing High-Titer Anti-Severe Acute Respiratory Syndrome Coronavirus 2 (SARS-CoV-2) Spike Protein IgG The American Journal of Pathology 2021 191 1 90 107 10.1016/j.ajpath.2020.10.008 33157066 PMC7609241

[24] Agarwal A Mukherjee A Kumar G Chatterjee P Bhatnagar T Convalescent plasma in the management of moderate covid-19 in adults in India: open label phase II multicentre randomised controlled trial (PLACID Trial) BMJ 2020 371 m3939 10.1136/bmj.m3939 33093056 PMC7578662

[25] Wibmer CK Ayres F Hermanus T Madzivhandila M Kgagudi P SARS-CoV-2 501Y.V2 escapes neutralization by South African COVID-19 donor plasma Nature Medicine 2021 27 4 622 625 10.1038/s41591-021-01285-x 33654292

[26] Joyner MJ Wright RS Fairweather D Senefeld JW Bruno KA Early safety indicators of COVID-19 convalescent plasma in 5000 patients The Journal of Clinical Investigation 2020 130 9 4791 4797 10.1172/JCI140200 32525844 PMC7456238

[27] Nguyen FT van den Akker T Lally K Lam H Lenskaya V Transfusion reactions associated with COVID-19 convalescent plasma therapy for SARS-CoV-2 Transfusion 2021 61 1 78 93 10.1111/trf.16177 33125158

[28] Joyner MJ Bruno KA Klassen SA Kunze KL Johnson PW Safety Update: COVID-19 Convalescent Plasma in 20,000 Hospitalized Patients Mayo Clinic Proceedings 2020 95 9 1888 1897 10.1016/j.mayocp.2020.06.028 32861333 PMC7368917

[29] Grisolia G Franchini M Glingani C Inglese F Garuti M Convalescent plasma for coronavirus disease 2019 in pregnancy: a case report and review American Journal of Obstetrics and Gynecology MFM 2020 2 3 100174 10.1016/j.ajogmf.2020.100174 32838270 PMC7332432

[30] Zhang B Liu S Tan T Huang W Dong Y Treatment With Convalescent Plasma for Critically Ill Patients With Severe Acute Respiratory Syndrome Coronavirus 2 Infection Chest 2020 158 1 e9 e13 10.1016/j.chest.2020.03.039 32243945 PMC7195335

[31] Salazar E Perez KK Ashraf M Chen J Castillo B Treatment of Coronavirus Disease 2019 (COVID-19) Patients with Convalescent Plasma The American Journal of Pathology 2020 190 8 1680 1690 10.1016/j.ajpath.2020.05.014 32473109 PMC7251400

[32] Khamis F Al-Zakwani I Al Hashmi S Al Dowaiki S Al Bahrani M Therapeutic plasma exchange in adults with severe COVID-19 infection International Journal of Infectious Diseases 2020 99 214 218 10.1016/j.ijid.2020.06.064 32585284 PMC7308750

[33] Xia X Li K Wu L Wang Z Zhu M Improved clinical symptoms and mortality among patients with severe or critical COVID-19 after convalescent plasma transfusion Blood 2020 136 6 755 759 10.1182/blood.2020007079 32573724 PMC7414593

